# Chemical Group Profiling, *In Vitro* and *In Silico* Evaluation of *Aristolochia ringens* on *α*-Amylase and *α*-Glucosidase Activity

**DOI:** 10.1155/2021/6679185

**Published:** 2021-06-04

**Authors:** J. B. Ahmad, E. O. Ajani, S. Sabiu

**Affiliations:** ^1^Department of Medical Biochemistry and Pharmacology, School of Basic Medical Sciences, Kwara State University, Malete, PMB 1530, Ilorin, Nigeria; ^2^Department of Biotechnology and Food Science, Faculty of Applied Sciences, Durban University of Technology, P.O. Box 1334, Durban 4000, South Africa

## Abstract

Diabetes mellitus (DM) has become a global scourge, and there is a continuous search for novel compounds as viable alternatives to synthetic drugs which are often accompanied by severe adverse effects. *Aristolochia ringens* is among the scientifically implicated botanicals effective in the management of several degenerative diseases including DM. The current study evaluated the inhibitory mechanism(s) of root extract of *A. ringens* on *α*-amylase and *α*-glucosidase *in vitro* and *in silico*, while its constituents were characterized using liquid chromatography-mass spectrometric technique. The extract had concentration-dependent inhibitory effect on the study enzymes, and the inhibition compared well with that of standard drug (acarbose) with respective IC_50_ values of 0.67 mg/mL (*α*-amylase) and 0.57 mg/mL (*α*-glucosidase) compared with that of the extract (0.63 and 0.54 mg/mL). The extract competitively and uncompetitively inhibited *α*-amylase and *α*-glucosidase, respectively. Of the identified compounds, dianoside *G* (−12.4, −12.5 kcal/mol) and trilobine (−10.0, −10.0 kcal/mol) had significant interactions with *α*-amylase and *α*-glucosidase, respectively, while magnoflorine and asiatic acid also interacted keenly with both enzymes, with quercetin 3-O-glucuronide and strictosidine showing better affinity towards *α*-glucosidase. These observations are suggestive of involvement of these compounds as probable ligands contributing to antidiabetic potential of the extract. While studies are underway to demystify the yet to be identified compounds in the extract, the data presented have lent scientific credence to the acclaimed *in vivo* antidiabetic potential of the extract and suggested it as a viable source of oral hypoglycaemic agent.

## 1. Introduction

Diabetes mellitus (DM) is a chronic, multifactorial set of metabolic diseases arising from deficiencies in insulin synthesis, insulin potency, or both which often manifests as severe hyperglycaemia. A recent report established that the global prevalence of DM is on the increase and expected to affect 693 million people by 2045 compared to figure (451 million) reported in 2017, if no suitable measures to curb the menace are embraced [1, 2]. Specifically, in Sub-Saharan Africa, approximately 15.9 million adults were diagnosed with DM over this period and projected to be around 41.6 million by 2045 [3]. Of the two major types of DM, type 2 DM (T2DM) is more common and accounts for around 90% of all diabetes cases worldwide [4] and has been managed through restoration of endogenous production of insulin, improving the sensitivity of insulin receptors, administration of oral antidiabetic drugs, and delaying glucose release from dietary starch by carbohydrate-degrading enzymes such as *α*-amylase (E.C. 3.2.1.1) and *α*-glucosidase (E.C. 3.2.1.20) [5, 6]. Alpha-amylase and *α*-glucosidase are the two principal carbohydrate metabolizing enzymes implicated in postprandial hyperglycaemia in T2DM. Specifically, *α*-amylase degrades starch to smaller components such as maltose, sucrose, and maltotriose, and the last stage in the degradation of these sugars is the hydrolysis of *α*-d-glucose residues from the nonreducing end of *α*-glucoside by *α*-glucosidase [7]. While increased activities of these enzymes have been linked with increased levels of plasma glucose, their inhibitions are often exploited in downregulating glucose absorption and subsequent lowering of postprandial glucose levels in T2DM sufferers [8, 9].

Despite the availability of many conventional *α*-glucosidase and *α*-amylase inhibitors, many are accompanied with life-threatening side effects including severe hypoglycaemia, weight gain, flatulence, and diarrhoea in addition to high cost, thereby reducing their effective application in the management of T2DM [10, 11]. Interestingly, the use of medicinal plants has become globally acceptable to complement the conventional treatment options, largely due to their relative availability, affordability, and safety profiles [6]. One of these medicinal plants is *Aristolochia ringens*.

*Aristolochia ringens* is indigenous to tropical America with prominent presence in roadside bush of West African countries like Sierra Leone, Ghana, Nigeria [12], and DR Congo [13]. The plant is commonly called “Dutchman's pipe,” with local names such as “Ako-igun” and “Dumandutsee” by the Yoruba and Hausa tribes of Nigeria, respectively. Different parts and extracts of the plant have been reported for their various medicinal applications. The root extracts of *A. ringens* have been reported as potent anticancer [14], antimicrobial [15], and anti-inflammatory [16] therapeutics, while the antidiarrheal activity of its stem-bark extract was reportedly remarkable [17]. In addition to its folkloric use as an oral hypoglycaemic agent, the antidiabetic potential of its root ethanolic extract through significant reduction in hyperglycaemia in streptozotocin-diabetic rats has also been reported [6]. Despite these reports about the antidiabetic potential of *A. ringens*, the exact mechanism involved remains elusive. Hence, the current study investigated its inhibitory effect on the specific activities of key enzymes implicated in the control of postprandial hyperglycaemia in T2DM, *in vitro* and *in silico* with a view to lending credence to its mechanism of hypoglycaemic action. Its chemical profile was also analyzed using liquid chromatography-mass spectrometric (LC-MS) technique.

## 2. Materials and Methods

### 2.1. Chemicals and Reagents

The alpha-glucosidase (from murine intestinal acetone powder (CAS No. I1630)), porcine pancreatic alpha-amylase (CAS No. A3176), dinitrosalicyclic acid (DNS) colour reagent, acarbose (CAS No. 56180-94-0), p-nitrophenyl-d-glucopyranoside (pNPG) (CAS No. 3767-28-0), and soluble starch (CAS No. 9005-84-9) were all products of Sigma–Aldrich Co. (St. Louis, MO, USA). The water used was glass-distilled and obtained from Medical Biochemistry and Pharmacology Laboratory, Kwara State University, Malete, Nigeria. Except otherwise stated, all other reagents and chemicals used are of high analytical grades.

### 2.2. Plant Material Collection and Authentication

The dried root of *Aristolochia ringens* used in this study was procured from a local market (Oja-oba), Ilorin, Kwara State, Nigeria, and authenticated (UILH/001/2019/1121) by Mr. Bolu Ajayi at the Department of Plant Biology, University of Ilorin, Ilorin, Nigeria.

### 2.3. Experimental Protocol

#### 2.3.1. Extract Preparation

Dried root of *A. ringens* was pulverized using Honda GX series multipurpose grinding machine (GX160, Mamtus Techshop, Lagos, Nigeria) before extraction of 150 g of the pulverized sample with 1500 ml of 70% ethanol for 24 h with intermittent shaking. The resulting filtrate was concentrated at 40°C over water bath (ASTM D6927, Milan, Italy).

### 2.4. *In Vitro* Enzyme Inhibition Assays

#### 2.4.1. Alpha-Amylase Inhibitory Assay

The method reported by Sabiu et al. [9] was adopted for this study. Concentrations ranging from 0.1 to 1.0 mg/mL of root ethanolic extract of *A. ringens* were used, and 500 *μ*L of each of the different concentrations and that of phosphate buffer (0.02 M, pH 6.9), containing porcine 0.5 mg/mL of *α*-amylase, was incubated (25°C, 10 min) in separate test tubes. Thereafter, 500 *μ*L of 1% starch solution, prepared in 0.02 M phosphate buffer (pH 6.9), was introduced to each test tube, and the resulting mixtures were further incubated at 25°C for 10 min before halting the reaction with DNS (1 mL). The test tubes were then boiled in a water bath for 5 minutes before each was cooled to room temperature. Absorbance measurement was done at 540 nm, the inhibition percentage was calculated, and nonlinear regression curve was used to extrapolate the half-maximal inhibitory concentration (IC_50_) value.

#### 2.4.2. Alpha-Glucosidase Inhibitory Assay

The method described by Elsnoussi et al. [18] was adopted in this assay. Varying concentrations (0.1–1.0 mg/ml) of both *A. ringens* extract and standard drug (acarbose) were made separately. Then, 50 *μ*L from the resulting stock solution was incubated (25°C for 10 min) after mixing with 100 *μ*L of 1.0 M *α*-glucosidase solution prepared in 0.1 M phosphate buffer (pH 6.9). This was immediately followed by addition of 50 *μ*L of 5 mM *p*NPG solution in 0.1 M phosphate buffer (pH 6.9) and further incubated at 25°C for 5 minutes. Absorbance was measured at 405 nm, and inhibition of *α*-glucosidase by *A. ringens* was calculated, and the IC_50_ was determined using nonlinear regression curve.

### 2.5. Inhibitory Kinetic Analysis

#### 2.5.1. Alpha-Amylase Kinetics

As previously reported [19], two separate sets of test tubes each containing exactly 100 *μ*L of *A. ringens* (at its IC_50_) and 0.02 M phosphate buffer (pH 6.9) were incubated for 10 minutes at 25°C with 100 *μ*L *α*-amylase solution. The reaction was initiated with the addition of starch substrate (S) concentrations (0.3–5.0 mg/ml) to both sets of test tubes, before the resulting mixture was further treated with 100 *μ*L DNS solution and boiled (100°C, 5 min). The sugar released from the reaction was estimated from maltose standard curve, after taking the absorbance (Beckman, DU 7400, USA). The resulting values were expressed as reaction rates (*v*), and double reciprocal plot [20] was used to determine the tentative mechanism of inhibitory effect of the extract on the activity of *α*-amylase from the obtained kinetic indices (Km and Vmax).

#### 2.5.2. Alpha-Glucosidase Kinetics

Preincubation of 50 *μ*L of *A. ringens* extract (at its IC_50_ value) or phosphate buffer (pH 6.9) was performed at a temperature of 25°C for 10 min, with 100 *μ*L of alpha-glucosidase solution in two sets of test tubes containing varying concentrations of *p*NPG (0.63–2.0 mg/mL; 50 µL). The reaction mixture was further incubated (10 min, 25°C) before it was halted by the introduction of 500 µL of Na_2_CO_3._ The released sugar was estimated colorimetrically from p-nitrophenol calibration curve. Lineweaver-Burk plot was employed to determine the nature of inhibition of *α*-glucosidase by *A. ringens* extract [20].

### 2.6. Liquid Chromatography-Mass Spectrophotometry (LC-MS) Data

The bioactive components of *A. ringens* ethanolic root extract were identified on a HP 1100 LC-MS system (Agilent Technologies, Santa Clara, CA) with a DAD-diode array detector and a quaternary pump, a Mass Selective Detector Ion Trap XCT mass spectrometer with an electrospray ionization interface (ESI). The injection volume of the extract was 0.5 *μ*L, loaded onto a C-18 column. The elution medium of two solvent systems A and B (90% acetate–water and 10% methanol, respectively) was a constant mobile phase made up of 10% B for 5 min, 10–100% B over 20 min, 100% B for 6 min, and reequilibration of the column, possessing a flow rate of 200 *μ*L/min. Spectra recordings were in the negative and positive ionization mode between m/z 50 and 1200. The bioactive compounds in the extract were identified through comparison of the fragment features with those on the National Institute Standard and Technology MS library (NIST-MS library).

### 2.7. Molecular Docking Analysis and Pharmacokinetic Properties Prediction

Molecular docking analysis was carried out following the procedures described by Yan et al. [21]. Protein Data Bank (PDB) (https://www.rcsb.org/) was searched for proper structural templates of the study enzymes, porcine pancreatic *α*-amylase (PDB Id : 1DHK; resolution 1.85 Å) and murine *α*-glucosidase (PDB Id : 5IEG; resolution 1.82 Å), and converted to pdbqt format with Open Babel, while the 3D structures of the study ligands (compounds and acarbose) were retrieved from Pubchem (https://pubchem.ncbi.nlm.nih.gov/). The structures obtained were optimized through addition of Gasteiger charges and nonpolar hydrogen atoms prior to docking into the binding pockets of the enzymes using AutoDock tool V4.2 (LGA, runs 100). The resulting complexes for each enzyme system with acarbose and the study compounds were ranked based on their docking scores. Porcine pancreatic *α*-amylase and murine *α*-glucosidase have frequently served as a model to evaluate the mechanism of inhibition of antidiabetic compounds with a fact that the two enzymes share high sequence similarities with those of human in their active core region [22, 23].

For the pharmacokinetic properties (absorption, distribution, metabolism, and excretion (ADME)) and drug-likeness of the most promising compounds obtained through docking, the SWISSADME server (http://swissadme.ch/index.php) was used.

### 2.8. Statistical Analysis

Except otherwise reported, the results of the inhibitory effect of the extract are presented as percentages (%), while other results are expressed as mean value ± standard error of the mean (SEM) of replicate (*n* = 3) experiment. Analysis of variance for differences between mean values was used to determine significant differences (*p* < 0.05) between each treatment using SAS statistical package (version 8.1, SAS Institute, Cary, NC, USA).

### 2.9. Results

**Figure 1:**
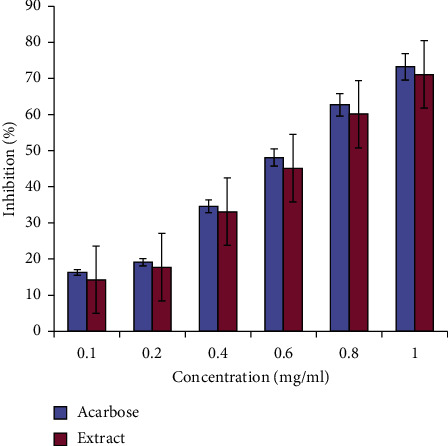
Inhibitory potential of *Aristolochia ringens* root extract on the activity of *α*-amylase. Bars (*n* = 3; mean ± SEM) at each level of concentration are not significantly (*p* > 0.05) different.

**Figure 2:**
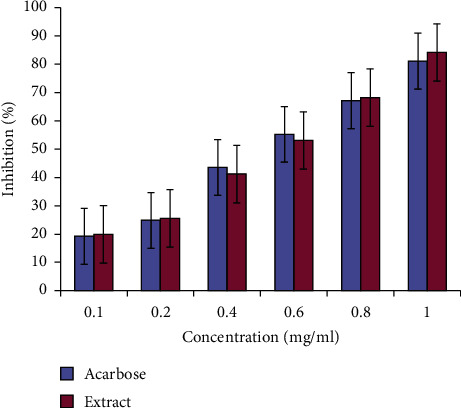
Inhibitory potential of *Aristolochia ringens* root extract on the activity of *α*-glucosidase. Bars (*n* = 3; mean ± SEM) at each level of concentration are not significantly (*p* > 0.05) different.

The inhibitory activity of ethanolic root extract of *A. ringens* on the activity of alpha-amylase and alpha-glucosidase was compared with that of the standard drug (acarbose) as shown in Figures 1 and 2 , respectively. The inhibition in each case was dose-dependent for both the extract and acarbose across the investigated dose levels. For both assays, the highest percentage inhibition occurred at the highest concentration for the extract and acarbose with no corresponding significant difference (*p* > 0.05) between the IC_50_ values of acarbose (0.67 mg/mL) and the extract (0.63 mg/mL) for alpha-amylase (Figure 1) and alpha-glucosidase (Figure 2) (0.57 and 0.54 mg/mL), respectively.

**Figure 3:**
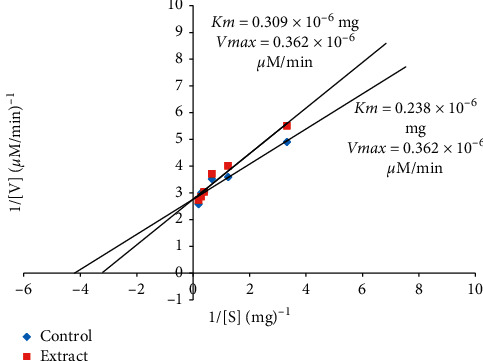
Double reciprocal plot of ethanolic root extract of *Aristolochia ringens* on the activity of *α*-amylase (competitive inhibition). Data represent mean ± SEM of three determinations.

**Figure 4:**
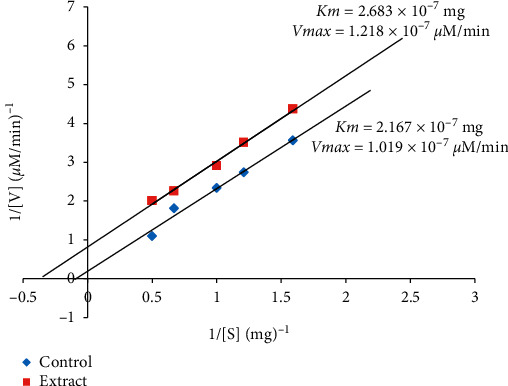
Double reciprocal plot of ethanolic root extract of *Aristolochia ringens* on the activity of *α*-glucosidase (uncompetitive inhibition). Data represent mean ± SEM of three determinations.

Figures 3 and 4 present the Lineweaver-Burk plots of ethanolic root extract of *Aristolochia ringens* showing competitive and uncompetitive inhibition on the activity of *α*-amylase and *α*-glucosidase, respectively. The kinetic parameters (Km and Vmax) obtained from the plot revealed a significant difference (*p* < 0.05) between the Km values of the extract (0.309 × 10^−6 ^mg) and control (0.238 × 10^−6 ^mg) but with similar Vmax value (0.362 × 10^−6^ *µ*M/min) (Figure 3). In sharp contrast to this observation, the inhibition pattern was uncompetitive for *α*-glucosidase as shown by the significant difference (*p* < 0.05) between the Km values of the extract (2.683 × 10^−7 ^mg) and control (2.167 × 10^−7 ^mg), as well as in their Vmax values (extract: 1.218 × 10^−7^ *μ*M/min; control: 1.019 × 10^−7^ *μ*M/min) (Figure 4).

**Figure 5:**
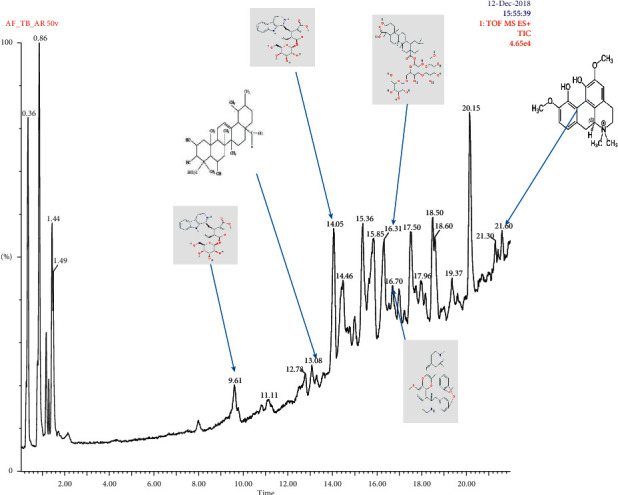
LC-MS chromatogram of root ethanolic extract of *Aristolochia ringens*.

The chemical group profile of ethanolic root extract of *Aristolochia ringens* as revealed by LC-MS analysis is presented in Table 1 and Figure 5. The chromatogram revealed the presence of 13 compounds including dianoside, trilobine, asiatic acid, magnoflorine, quercetin-3-*O-*glucuronide, and strictosidine with retention times at 16.31, 16.70, 13.08, 21.60, 9.61, and 14.05 minutes, respectively (Figure 5). Other compounds identified include aristolochic acid, corosolic acid, neoasarinin, quercetin 3-p-coumaroylglucoside, phenyl-*β*-D-glucopyranoside, and two other unidentified compounds (Table 1).

**Figure 6:**
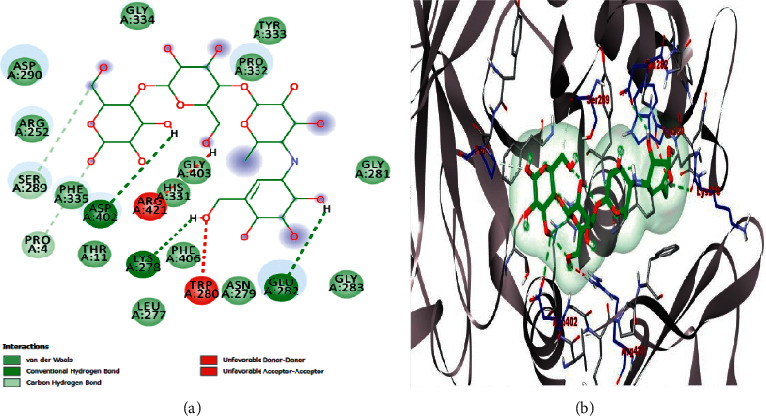
Images showing (a) 2D and (b) 3D interaction plots of acarbose and alpha-amylase.

**Figure 7:**
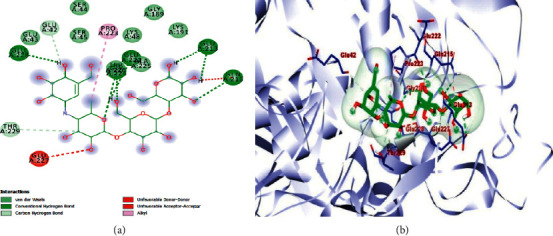
Images showing (a) 2D and (b) 3D interaction plots of acarbose and alpha-glucosidase.

**Figure 8:**
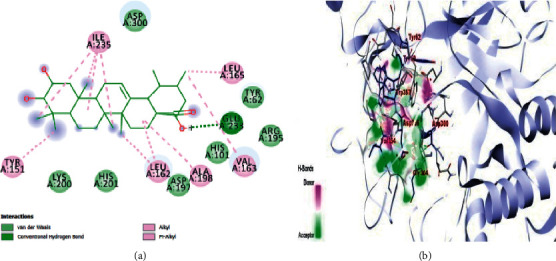
Images showing (a) 2D and (b) 3D interaction plots of dianoside G and alpha-amylase.

**Figure 9:**
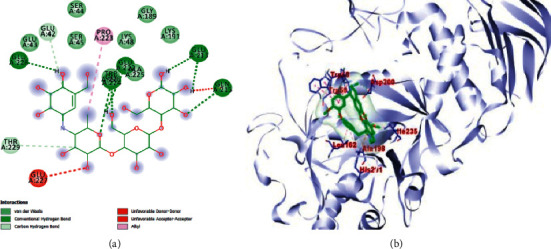
Images showing (a) 2D and (b) 3D interaction plots of trilobine and alpha-amylase.

**Figure 10:**
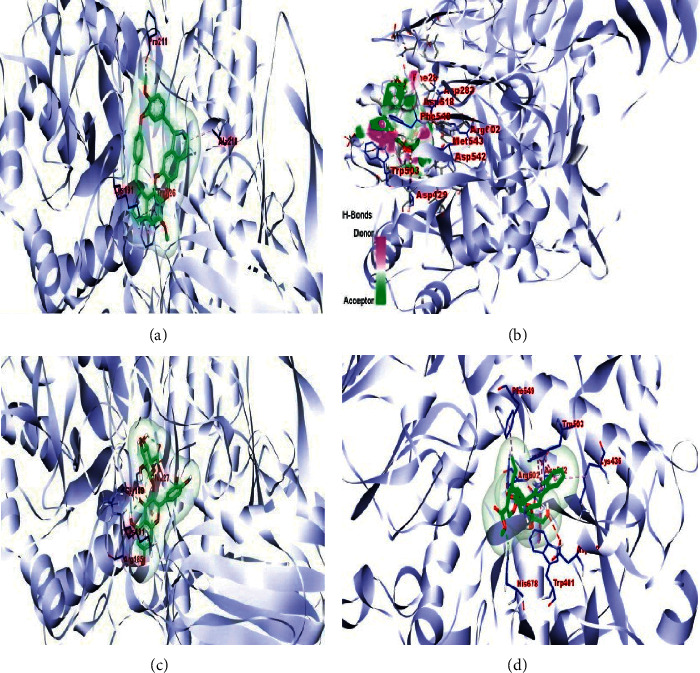
Images showing 3D interaction plots of alpha-glucosidase with (a) trilobine, (b) dianoside G, (c) quercetin 3-O-glucuronide, and (d) strictosidine

**Figure 11:**
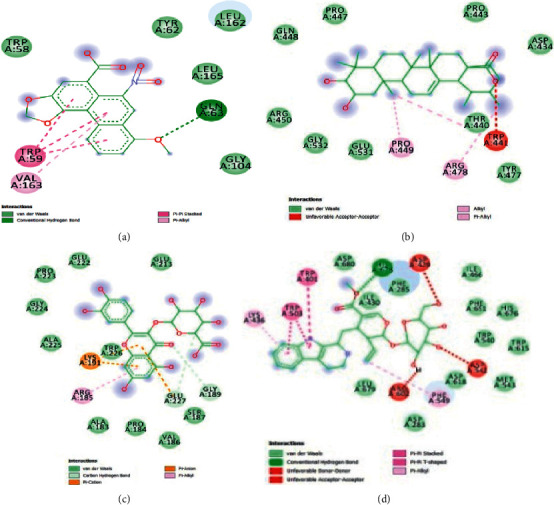
Images showing 2D interaction plots of alpha-glucosidase with (a) trilobine, (b) dianoside G, (c) quercetin 3-O-glucuronide, and (d) strictosidine

The results obtained with respect to the docking properties of the identified compounds against *α*-amylase and *α*-glucosidase are shown in Table 2. Binding interactions of the study compounds with the enzymes revealed that dianoside *G* and trilobine had higher binding energy scores of −12.4 and −10.0 Kcal/mol, respectively, than acarbose (−7.8 Kcal/mol) with *α*-amylase, as against higher values ranging from −8.0 Kcal/mol (asiatic acid) to −12.5 Kcal/mol (dianoside G) than acarbose with *α*-glucosidase (Table 2). The interaction plots of the docked complexes and amino acid residues at the binding pockets of the enzymes are presented in Figures 6–11 . Dianoside *G* had favourable interaction through hydrogen bonding with Gly 304, Val 354, Asp 306, and cation-pi interaction with Trp 357 in the active site of *α*-amylase. Other hydrophobic interactions such as carbon-hydrogen and van der Waals bonds were also found between dianoside *G* and residues Asp 300, Tyr 62, and Trp 59 of the enzyme (Figure 8). Similarly, trilobine interacted with residues Trp 58, Trp 59, Asp 300, Leu 162, Ala 198, Ile 235, and His 201 at the binding pockets of *α*-amylase predominantly through noncovalent bonding, while its interaction with residues Trp58, Trp59, and His201 is through pi-pi T-shaped, pi-pi stacked, and pi-cation interactions, respectively. Other residues such as Asp300, Leu162, Ile235, and Ala198 on the enzyme interacted with trilobine through C–H bonds, pi-pi stacking, pi-pi, and pi-pi T-shaped interactions (Figure 9). For glucosidase, trilobine interacted favourably with residues Pro 211, Ala 218, Trp 26, and Lys 191 at the binding pocket. It also interacted via van der Waals forces with residues Pro211 and Trp226 and through pi-cation and pi-pi stacked with Lys191 and Ala218, respectively (Figure 10).On the other hand, dianoside *G* was found to interact with Phe 28, Phe 549, and Trp 503 through H-bonds and pi bond interactions, while van der Waals forces and C–H bonds stabilized its interaction with residues Asp 429, Asp 542, Met 543, Arg 602, Asp 618, and Asp 283 (Figures 10 and 11).

Except for trilobine that is hydrophobic and passed the drug-likeness test as predicted by Lipinski's rule of five, acarbose and dianoside *G* were lipophilic (iLogP < 5) (Table 3). Similarly, compared to other compounds, trilobine was predicted with a high absorption rate in the gastrointestinal tract (GIT) and with higher degree of synthetic accessibility. For bioavailability, dianoside *G* compared well with acarbose, while trilobine had a higher score of 0.55 (Table 3).

## 3. Discussion

*α*-Amylase and *α*-glucosidase remain the two major enzymes of focus in the regulation of postprandial glucose level in combating T2DM [24]. These enzymes are involved in the hydrolysis of dietary starch to simple sugar such as glucose, with concomitant elevation of systemic concentration of glucose. Accordingly, strategic inhibition of the two enzymes using either medicinal plants or plant-derived compounds has been shown to downregulate glucose absorption and offer effective control of postprandial hyperglycaemia in T2DM [25]. In this study, the observation on the inhibitory activity and that the IC_50_ values of the studied extract against *α*-amylase and *α*-glucosidase compares well with that of the standard drug (acarbose) and could be suggestive of potential hypoglycaemic attribute of the extract.

A further probe revealing that the extract competitively inhibits *α*-amylase could be supportive evidence on the manner of inhibitory influence of the extract on the enzyme, suggesting that the main antidiabetic constituents of the extract could compete and interact with the enzyme at the expense of its normal substrate. Such interaction will allow for reduction in the pace of conversion of starch to free glucose, which is important for postprandial hyperglycaemia control. This observation on the inhibitory effect of the *A. ringens* extract is consistent with previous reports [19, 26, 27] where plant extracts were found to be potent inhibitors of *α*-amylase. Similarly, the mode of inhibition of *α*-glucosidase by the extract is uncompetitive as revealed by its reduced Km and Vmax values. This could be suggestive of a higher attraction of the enzyme for the extract than its normal substrate and such preference will potentiate the extract to delay/modulate subsequent carbohydrate hydrolysis as earlier reported in other studies [28–30].

Docking of ligands (potential inhibitors) against known enzymes allows for better appreciation of the nature of interactions between the two entities [31]. This was undertaken in the present study to have a better understanding of the interaction between the identified constituents of the extract and both *α*-amylase and *α*-glucosidase. The docking scores obtained suggest that dianoside *G* and trilobine had better affinity for both *α*-amylase and *α*-glucosidase. The two compounds interacted better with amino acids at the binding pockets of the enzymes to achieve effective inhibition. Dianoside *G* was stabilized in the active pocket of *α*-amylase through interactions established by hydrogen bonds, cation-*π*, and other hydrophobic interactions such as van der Waals forces and C–H bonds. These bonds and interactions allow for the proper positioning of dianoside *G* with the catalytically important amino acid residues and could account for the affinity displayed. Similarly, the catalytic residues in the active pocket of *α*-glucosidase favourably interacted with dianoside *G* to achieve maximal affinity and stability. While the bulk of interaction was stabilized by van der Waals forces and C–H bonds (with Asp 429, Asp 542, Met 543, Arg 602, Asp 618, and Asp 283), H-bonds and *π*-*π* interactions stabilized dianoside G's interaction with the amino acid residues Phe 28, Phe 549, and Trp 503.

The favourable interaction of trilobine with the catalytically important residues of amylase is due to *π*-*π* stacking, *π*-*π* T-shaped, and *π*-alkyl interactions of trilobine with amino residues Trp58, Trp59, and His201, respectively, on the active cleft of amylase. This interaction is further enhanced by van der Waals and C–H bonds of trilobine with Ala198, Asp300, Leu162, and Ile235. Additionally, a number of hydrophobic and noncovalent interactions such as *π*-cation (with residue Lys191) and *π*-*π* stacking (with residue Ala218), together with van der Waals forces (with residues Pro211 and Trp226), are responsible for the affinity and subsequent stability of trilobine in the active pocket of glucosidase. Such bonds and interactions have been reported to stabilize amylase- and glucosidase-ligands relationships in previous studies [32, 33]. The observed favourable interaction could account for the lower binding energies (-10.0 and -10.0 kcal/mol) for amylase and glucosidase, respectively. Additionally, some of the amino acids (Tyr155, Tyr82, Asp340, and Asp206) in the active pockets of amylase and glucosidase in this study have been previously identified as the common amino acids stabilizing the interactions of the two enzymes with different ligands of antidiabetic importance [34–36].

While magnoflorine (−7.3, −8.2 kcal/mol) and asiatic acid (−7.5, −8.0 kcal/mol) also had significant interactions with *α*-amylase and *α*-glucosidase, respectively, quercetin 3-O-glucuronide (−8.9 kcal/mol) and strictosidine (−8.5 kcal/mol) were more active against *α*-glucosidase. Interestingly, asiatic acid and magnoflorine have earlier been reported for their hypoglycaemic potentials [37, 38], while quercetin 3-O-glucuronide has been shown to ameliorate insulin resistance through inhibition of reactive oxygen species [39], and these could be further attestation to the hypoglycaemic effect of *A. ringens*.

Lipinski's rule examines both the physical and chemical properties of a compound to ascertain its safety as an orally active drug [40]. The result of this study revealed that trilobine is a probable better therapeutic agent than acarbose and dianoside *G* that failed two of the rules: number of H-bond acceptors and H-bond donors higher than 10 and 5, respectively. The absorption of drugs in the GIT is crucial to maintaining optimal systemic concentration and getting to the target site with required concentration for maximum therapeutic effects [41]. Again, only trilobine showed high absorption tendency, with low absorption rate predicted for acarbose and dianoside G. Higher bioavailability score means high rate of absorption of a drug and the concentration of unchanged drug that reaches the site of action [42]. The higher bioavailability value for trilobine could mean that it will have higher concentrations at the site of action and eventually exert significant effect when considered as therapeutic agents.

## 4. Conclusion

Judging by the data presented in this study, it could be inferred that the *in vitro* competitive and uncompetitive inhibitory effect of root extract of *A. ringens* on *α*-amylase and *α*-glucosidase, respectively, could be attributed to the interactions of the enzymes with its active constituents such as dianoside *G*, trilobine, asiatic acid, magnoflorine, quercetin 3-O-glucuronide, and strictosidine as revealed by the *in silico* analysis. This study has therefore corroborated previous *in vivo* studies on the antidiabetic potential of the extract by lending scientific credence to one of its probable mechanisms of action through effective control of postprandial hyperglycaemia. Effort is ongoing to identify the two unknown compounds, with all available information pointing to their novelty. Therefore, isolation and purification of active compounds already identified in this study could avail novel therapeutic agent for the management of T2DM.

## Figures and Tables

**Table 1:** Chemical profile of ethanolic root extract of *Aristolochia ringens*.

S/N	RT (min.)	*m/z*	Fragment ions	Compound
1	1.42	360 [*M* + 1]^+^	325 (34), 163 (37), 146 (68), 198 (15)	Aristolochic acid I
2	9.61	611 [*M* + 1]^+^	303 (97), 465 (70), 197 (63), 372 (30)	Quercetin-3-*O-p*-coumaroyl glucoside
3	10.79	566 [*M* + 1]^+^	548 (83), 287 (40), 449 (34)	Unidentified
4	13.08	489 [*M* + 1]^+^	457 (51), 169 (17), 214 (11), 391 (9)	Asiatic acid
5	14.86	473 [*M* + 1]^+^	457 (50), 169 (17), 214 (11), 391 (9)	Corosolic acid
6	13.28	501[*M* + Na]^+^	401 (25), 169 (15)	Quercetin-3-*O-*glucoronide
7	14.05	531 [*M* + 1]^+^	485 (74), 453 (43), 435 (5)	Strictosidine
8	16.31	485[*M* + Na]^+^	473 (43), 419 (38), 183 (38)	Dianoside
9	16.70	563 [*M* + H]^+^	505 (80), 487 (54), 473 (28)	Trilobine
10	18.16	387[*M* + Na]^+^	267 (15), 183 (10)	Neoasarinin
11	18.48	274 [*M* + 1]^+^	217 (5), 169 (4)	Unidentified
12	20.15	478[*M* + Na]^+^	304 (11), 415 (8), 253 (5)	Phenyl-*β*-D-glucopyranoside
13	21.60	343 [*M* + 1]^+^	240 (56), 332 (20), 169 (11)	Magnoflorine

RT = retention time; *m/z* = mass to charge ratio.

**Table 2:** Docking properties of bioactive compounds from *A. ringens* extract against *α*-amylase and *α*-glucosidase.

S/N	Ligand molecules	*α*-Amylase		*α*-Glucosidase	
		Scores (kcal/mol)	Amino acids involved in the interaction	Scores (kcal/mol)	Amino acids involved in the interaction
1	Asiatic acid	−7.5	Trp 58, Trp 59, Ala 198, Val 163, Leu 162, His 101,	−8.0	Ala 218, Gly 216, Gln 215, Ile 214, Gly 224, Trp 226, Pro 211, Glu 227
2	Magnoflorine	−7.3	Gln 63, Trp 59	−8.2	Pro 116, Try 113, Tyr 563, Thr573, Pro 515, Tyr 57
3	Phenyl-*β*-d-glucopyranoside	−4.6	Pro 4, Phe 335, Pro 332, Gly 403, Asp 402, Arg 398, Arg 421	−5.0	Phe 852, Pro 597, Ala 381, Thr385
4	Aristolochic acid I	−5.9	Gln 63, Val 163, Trp 59	−7.1	Leu 388, Ala387, Arg 610
5	Corosolic acid	−2.5	Val 163, Leu 165, Leu 162, Ala 198, Ile 235, Tyr 151, Glu 233, Ala 198	−8.2	Arg 478, Trp 441, Pro 449
6	Dianoside G	−12.4	Gly 304, Val 354, Asp 306, Asp 300, Trp 357, Trp 59, Tyr 62	−12.5	Asp 429, Trp 503, Asp 542, Met 543, Arg 602, Phe 549, Asp 618, Asp 283, Phe 28
7	Neoasarinin A	−6.3	Pro 4, Arg 10, Arg 252, Asp 290, Phe 335, Ser 289, Gly 403, Asp 402, Gln 404, Arg 421	−7.0	His 676, Asp 29, Trp 401, Arg 602, Asp 613, Met 543, Trp 503, Phe 549
8	Quercetin 3-(3-p-coumaroylglucoside	−7.0	Arg 252, Asp 290, Ala 3, Pro 4, Pro 332, Pro 405, Gln 404, Asp 402, Arg 421, Arg 398	−7.2	Thr22, Gly 227, Arg 185, Lys 191, Trp 26, Gly 234
9	Quercetin 3-O-glucuronide	−7.0	Arg 252, Gly 334, Phe 335, Pro 332, Gly 403, Asp 402, Arg 398, Arg 421	−8.9	Pro 223, Ala 218
10	Strictosidine	−5.5	Asp 402, Gln 404, Arg 421, Pro 405, Phe 406, Arg 398, Pro 332, Phe 335, Gly 334, Arg 252	−8.5	His 678, Trp 401, Lys 436, Arg 602, Asp 542, Trp 503, Phe 549
11	Trilobine	−10.0	Trp 58, Trp 59, Asp 300, Leu 162, Ala 198, Ile 235, His 201	−10.0	Pro 211, Ala 218, Trp 26, Lys 191
12	Acarbose^*∗*^	−7.8	Arg 421, Asp 402, Lys 278, Trp 280, Glu 282, Ser 289, Pro 4	−7.4	Glu 222, Glu 42, Pro 223, Gln 215, Gly 214, Glu 218, Glu 228, Glu 227, thr 229

**Table 3:** Pharmacokinetic properties of dianoside *G*, trilobine, and acarbose.

Properties	Acarbose	Dianoside G	Trilobine
Molecular formula	C_25_H_43_NO_18_	C_48_H_76_O_20_	C_35_H_34_N_2_O_5_
Molecular weight (g/mol)	645.60	973.11	562.65
Bioavailability score	0.17	0.11	0.55
Water solubility	High	Moderate	Poor
Lipophilicity (ilogP)	0.63	2.49	4.66
GIT absorption	Low	Low	High
BBB-permeability	No	No	No
Hydrogen bond acceptors	19	20	7
Hydrogen bond donors	14	12	1
Lipinski's rule	No	No	Yes
Synthetic accessibility	7.34	9.91	6.60

## Data Availability

Relevant data have been included in the manuscript and can also be obtained upon request to the corresponding author.
